# Effect of Virtual Reality on Balance Function in Children With Cerebral Palsy: A Systematic Review and Meta-analysis

**DOI:** 10.3389/fpubh.2022.865474

**Published:** 2022-04-25

**Authors:** Wei Liu, Yuanyan Hu, Junfeng Li, Jindong Chang

**Affiliations:** ^1^School of Physical Education, Xuzhou Kindergarten Teachers College, Xuzhou, China; ^2^School of Mathematics and Statistics, Yunlin Normal University, Yunlin, China; ^3^Ministry of Sports, Shandong Technology and Business University, Yantai, China; ^4^Institute of Motor Quotient, Southwest University, Chongqing, China

**Keywords:** virtual reality, cerebral palsy, balance, interactive games, systematic review

## Abstract

Virtual Reality (VR) therapy is popular in treating children with Cerebral Palsy (CP) as a new technology for rehabilitation. Nevertheless, no substantial evidence supporting VR therapy promotion has been developed to date. This study aimed to investigate the effects of VR therapy on balance in children with CP. We conducted a systematic search in PubMed and Web of Science (updated to December 30, 2021). The systematic review and meta-analysis included all randomized controlled trials that included children with CP. A total of 18 RCT studies were eligible for inclusion in the systematic review, and meta-analysis was performed on 16 of them. Results showed that the VR intervention was beneficial for balance (SMD 0.47 [95% CI, SD 0.28, 0.66]). We concluded that VR therapy interventions for children with CP have positive effects. However, cautious implementation is needed in clinical applications.

## Introduction

Cerebral Palsy (CP) is a neurological disorder caused by non-progressive brain injury and developmental defects (1). The main manifestations are central motor deficits and postural disorders, which may also be accompanied by developmental delay, epilepsy, perceptual impairment, language disorders, and cognitive behavior abnormalities (2). According to the World Health Organization, the incidence of CP in developed countries is 0.2–0.3%, and the incidence of CP in China is about 0.248% (2–4). As reported, there are 6 million children with cerebral palsy in China, with an average annual increase of 50,000, which has become a severe problem in public health (5). CP is a significant cause of physical disability in children, and late damage to the central nervous system in children with CP can cause secondary injuries such as limb spasticity, muscular atrophy, skeletal deformities, muscle weakness, and developmental coordination disorders, which limit the child's mobility and thus affect the development of gross motor skills (6, 7). Studies have shown that failure to promptly identify and remedy impairments in the development of gross motor skills may lead to motor deficits (8). Current data show that 72–91% of children with CP have limitations in activities of daily living (ADLs) such as outdoor walking, stair climbing, and self-care activities (9). Restrictions in mobility and self-care are often associated with lower extremity impairment, making lower extremity function important for ADLs.

Motor skill training or rehabilitation is commonly associated with improvements in balance and walking ability of the lower limbs (10). In contrast, the quantity and quality of training are essential to promote plasticity and functional recovery of the child's brain (11). Therefore, developing a practical intensive training or rehabilitation program requires consideration of time and intensity (12, 13). Some studies have demonstrated that traditional center-based CP rehabilitation programs (e.g., hospitals, gyms, sports centers) positively affect children with CP with 30–45 min sessions per day, which seems to be necessary for neuroplasticity (14–17). Traditional center-based approaches in physical therapy, such as group therapy and therapist-assisted therapy, target children with certain types of CP in a face-to-face manner and can enhance communication between children with CP and their parents (18). However, center-based health care systems are often unable to provide weekly interventions for children with CP because of the time-consuming and expensive costs (19). Hence, it is necessary to find cost-effective physical therapies that can help children with CP long enough intensive training. Recently, family interactive training has been positive for the rehabilitation of children with cerebral palsy, improving balance (20). Consequently, home-based task-oriented exercise effectively complements center-based occupational therapy and physiotherapy to ensure more intensive and sustained exercise for children with CP (2, 21).

Virtual Reality (VR) therapy is a recently popular assistive technology in the rehabilitation of children with CP (22). Its characteristic is that people can immerse themselves in a non-physical world through 3D displays at home (23). An active video game is used and facilitates the systematic practice of functional movement and multisensory feedback (19). This immersive experience is in a safe and enjoyable environment, which may appeal to children, including those with CP (24). Some studies showed that VR-based rehabilitation facilitated perceptual training and task completion in a virtual environment similar to reality but with a higher predictability and activity control (19, 24, 25). Indeed, active video games promote functional activities with multisensory demands, active muscle stretching, and motor training that challenge postural stability, creating favorable conditions for children with CP (19, 26). Some studies showed that VR-based therapies provided visual perceptual stimuli generated by dynamic changes in the environment, which facilitated controlled exercise in children with CP (27–30). When children play games, the actions involved, such as laughing, gesticulating, and screaming, could enhance bioelectrical signals in the brain (31). Moreover, home-based VR therapy could enhance the somatic experience of these games. Factors such as the duration, intensity, and repetition of children's activities may improve their condition and facilitate the recovery of motor function in children with CP (32, 33). Compared to traditional center-based rehabilitation, home-based VR therapy has the advantages of small space (traditional centers require dedicated rehabilitation) and low cost (traditional center-based rehabilitation requires experienced therapists) (34, 35). Therefore, children with CP that are generally reluctant to receive traditional therapy tend to prefer VR therapy (19, 36), which will contribute to the motor skill development of children with CP. Home Virtual Reality GAME (VRG) therapy has become increasingly crucial for the rehabilitation of children with CP due to family economic reasons.

**Figure 1:**
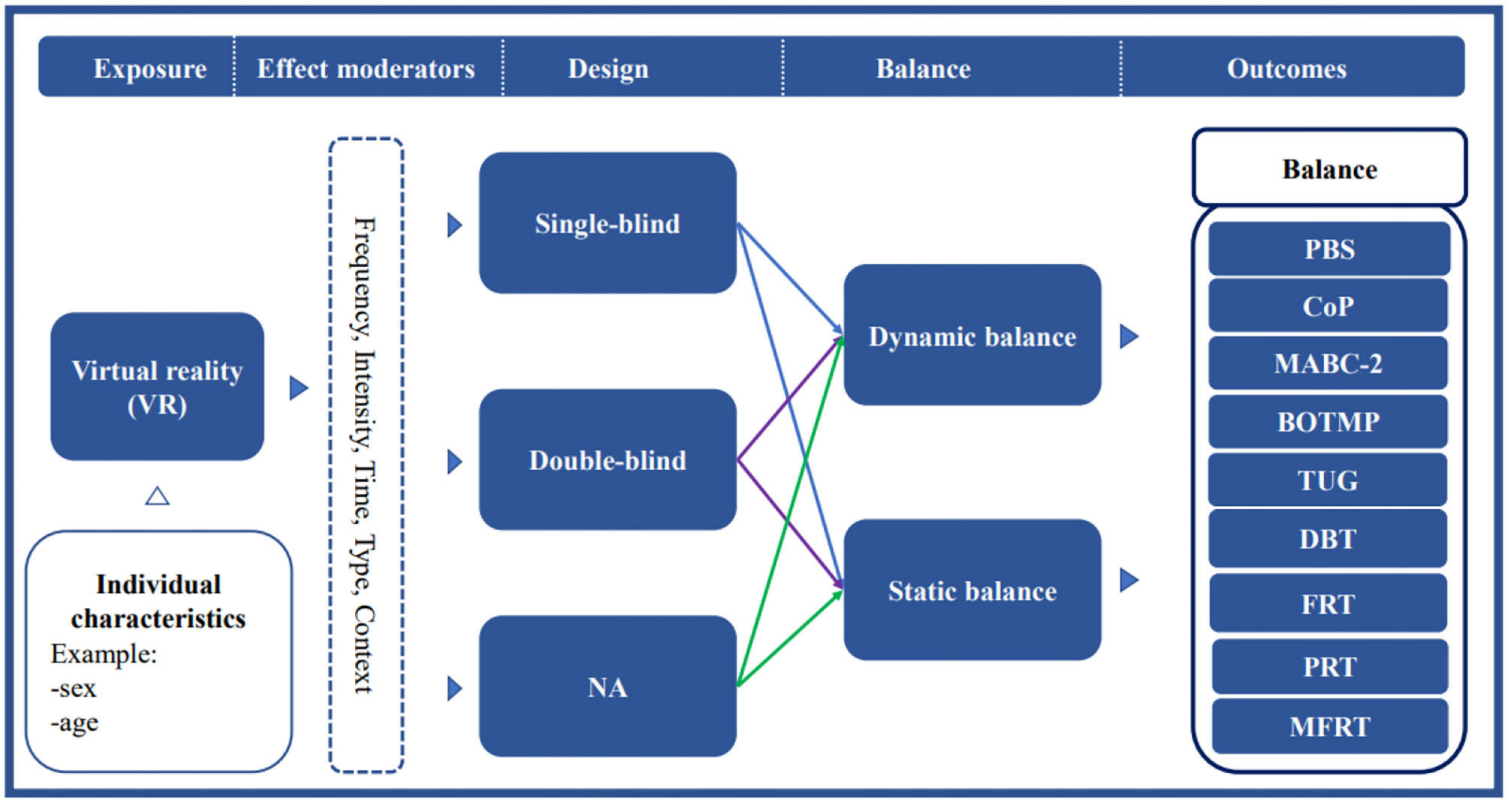
A conceptual model of VR therapy intervention for balance function.

Review studies on VR therapy for children with CP showed that VRG therapy interventions could improve the development of gross motor skills, including strength, balance, coordination, and other physical qualities in children with CP (2, 37, 38). Meta-analysis of VRG on upper limb motor skills also found that VRG in a VR setting is a feasible instrument for improving motor skills in children with CP (38). Although previous review studies showed improvements in gross motor skills and upper limb skills in children with CP, the evidence for their assessment was limited by studies including randomized controlled trials (39, 40). Recent studies by Sajan et al., Pin et al., and Jha et al. did not support the hypothesis that VR therapy is more effective than physical therapy for balance in children with CP (41–43). To date, there is no strong evidence from studies showing the effectiveness of VR therapy on balance in children with CP. As such, this study aimed to explore the effects of VR therapy on balance in children with CP using a meta-analysis. Consequently, a conceptual model of the effects of VR therapy intervention for balance function is needed to analyze the strengths or weaknesses of typologies and methodologies (Figure 1). Additionally, the effects of the VRs intervention program (including single intervention time, intervention frequency, intervention period, and total intervention time) on the gross motor skills of children with CP were further determined. Thus, an essential theoretical basis for the effect of VR-based therapy on the balance ability of children with CP was established, which provides a vital decision basis for clinical rehabilitation staff.

## Method

### Search Strategy and Screening

A systematic review and meta-analysis were conducted and implemented following the Preferred Reporting Items for Systematic Reviews and Meta-Analyses guidelines (PRISMA) (44). The online databases of PubMed and Web of Science were used to search for relevant studies up to December 30, 2021. The following search terms were used in the PubMed Mesh term and title abstract search terms. “video games” or “serious games” or “virtual reality” or “interactive games” or “VR” and “cerebral palsy.” The same search terms as PubMed were used in the Web of Science Title/Abstract/Keyword search term. The search results were processed in Endnote Library X7, and duplicates were removed. Two independent reviewers assessed the full-text article (WL and YH). A third reviewer (JL) was adjudicated if no consensus was achieved. The reference lists of relevant full-text articles were manually searched to identify all relevant references.

### Selection Criteria

The following criteria were met for inclusion in the study. (1) Participants were diagnosed with CP and their age was no more than 18 years. CP was defined as permanent impairments in motor and postural development that were often accompanied by insensitivity, cognition, communication, perception, and behavior problems. Participants had limitations in daily activities. (2) VR was used as a therapeutic intervention. VR was defined as a concept that uses real-time interaction with the patient and feeds back their motor patterns or performance knowledge acquired through visual, auditory, proprioceptive, vestibular, or olfactory stimuli. Visual stimuli could be displayed on a monitor, flat-screen, projection screen, or head-mounted device. VR could potentially produce environments with realistic-looking objects, or it could be a game environment. (3) Randomized controlled intervention studies with pre- and post-experimental performance were assessed using a balanced outcome measure. (4) Effects on balance were measured by motor performance scales or test instruments used pre-and post- VR.

The exclusion criteria were: (1) case reports, (2) non-peer-reviewed publications, (3) conference abstracts, (4) non-English language publications, (5) VR as an intervention supplement, (6) studies that included patients with comorbidities, and (7) studies that included other interventions that affected VR balance performance.

### Data Collection

Data and information were collected in the included studies, including age, gender, number of participants, inclusion and exclusion criteria. The following outcomes were recorded for the VR experimental group: type of VR intervention, blinding of the RCT design, and balanced outcome measures. Two authors independently extracted this information from the included articles (38).

### Qualitative Analysis

The Physiotherapy Evidence Database (PEDro) scale was used to assess the methodological quality of randomized controlled trials (RCTs) to assess the risk of bias (45, 46). The PEDro scale included 11 items, one associated with eligibility criteria (item 1, not scored), eight reflecting internal validity (items 2–9), two representing statistical comparisons between groups (item 10), and a measure of variability (item 11). Results of two independent reviewers' assessments were compared, and consistent decisions were made to ensure the accuracy and completeness of data extraction. A third reviewer resolved any persistent discrepancies and discussed further as necessary.

### Data Analysis

Outcome measures on balance were extracted from the included studies and reported. Extracted data from the article included data related to mean change from baseline and Standard Deviation (SD) and sample size for the VR and control groups. The authors were contacted first if exact values cannot be extracted from the article. If contacting the authors was unsuccessful, alternatively, calculations based on the data provided in the article would be considered. When SD of change scores was not available in the study, the formula described in the Cochrane Handbook was used. For this formula, correlation coefficients were extracted from the available literature of the clinical trials used. All outcomes were included if a study used different outcome measures to determine balance. In these studies, the total number of participants was divided by the number of tests used (according to the Cochrane Handbook) to calculate heterogeneity. Random-effects models were used even when heterogeneity was low because of the different clinical trials used in the included articles: heterogeneity only claimed that the results included in the analysis were not statistically heterogeneous and did not consider differences in the use of different clinical trials (38). Standardized mean differences (SMD) were used as effect measures because sometimes outcomes measured using different instruments were used to determine the same outcome (38). Effect sizes were determined using the rules of Cohen 1988 in this study (47). Review Manager 5.3 was used to perform a meta-analysis.

## Results

### Identification of Studies

**Figure 2:**
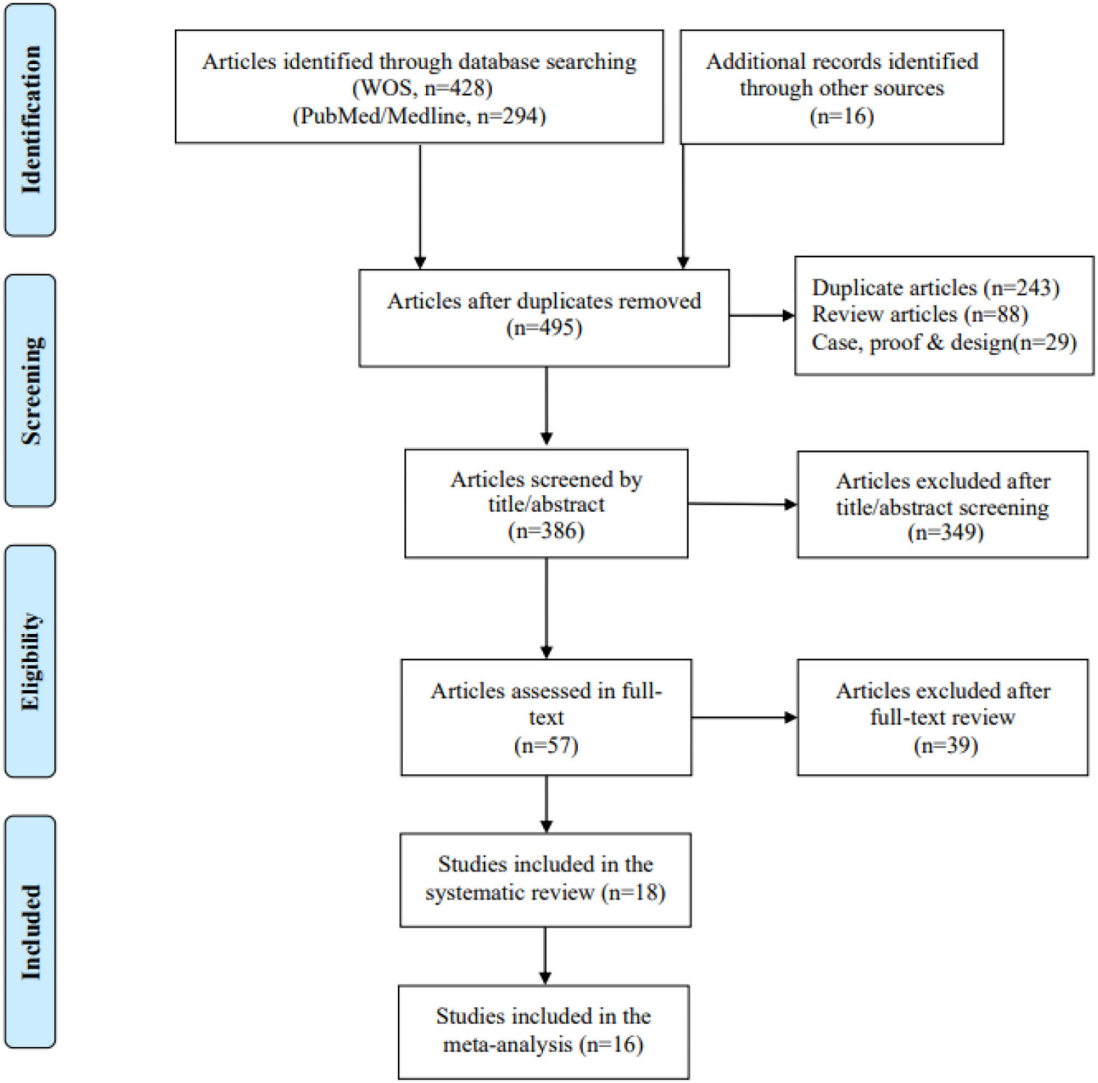
PRISMA flowchart of included and excluded studies.

A total of 493 articles were retrieved after searching PubMed and WOS databases to remove duplicate data, of which 57 full-text articles were checked for eligibility (Figure 2). The 18 RCT studies were considered eligible for inclusion in the systematic review, of which 16 studies were obtained with valid data for meta-analysis.

### Description of Included Studies

A total of 474 patients were included in the 18 studies (Table 1). The age range of participants was 4–18 years. The participants in one study included only Gross Motor Function Classification System (GMFCS) Level I (48), four studies included GMFCS Levels I-II (19, 49–51), eight studies included GMFCS Levels I-III (52–57), one study included GMFCS Levels I-IV (41), and four studies included GMFCS Levels III (58) or III-IV (42, 62, 63). All studies measured balance using at least one instrument, and five studies used two or more instruments to assess patients' balance (9, 53–56). Eight studies used the PBS assessment tool (41, 43, 51–53, 55, 57, 59), five studies used the CoP Kinematics system (balance force plate) (19, 41, 48, 50, 53), three studies used the TUG (54–56), two studies used the BOTMP (9, 49), and one study used the MABC-2 (58), FRT (56), MFRT (60), PRT (42) and DBT (54).

**Table 1:** The characteristics of the inclusion studies.

**References**	**Age (years)**	**Numbers total/male**	**Duration**	**Total (min)**	**RCT Design**	**VR Intervention**	**Balance**	**GMFCS**	**Location**	**Outcome measure**
AlSaif and Alsenany (58)	Range 6–10	40/NA	20 min/d*7 d/wk*12 wk	1,680	NA	Nintendo Wii fit game	DB	III	Home	Balance: MABC-2
Arnoni et al. (19)	Mean: 10 ± 3	9/5	45 min/d*2 d/wk*8 wk	720	single-blind	Xbox 360 Kinect sensor	SB	I–II	SL	Balance: CoP
Chen et al. (49)	Range 6–12	28/19	40 min/d*3 d/wk*12 wk	1,440	NA	Virtual cycling system with interactive workouts	DB	I–II	Home	Balance: BOTMP
Cho et al. (52)	VR:10.2 ± 3.4 CG:9.4 ± 3.8	18/NA	30 min/d*3 d/wk*8 wk	720	single-blind	Nintendo Wii jogging program	DB	I–III	SL	Balance: PBS
Decavele et al. (59)	Range 6–15	32/NA	45 min/d*2 d/wk*12 wk	1,080	single-blind	MS Kinect for Windows and Nintendo Wii balance board	DB	III–IV	SL	Balance: PBS
Gatica-Rojas et al. (50)	Range 7–14 Mean:10.4	32/19	25 min/d*3 d/wk*6 wk	450	no blinding	Wii Fit Plus with the Nintendo Wii Balance Board	SB	I–II	SL	Balance: CoP
Hsieh (53)	Mean:7.33 ± 1.31	40/29	45 min/d*3 d/wk*12 wk	1,620	NA	Customized PC gaming	SB/DB	I–III	SL	Balance: CoP/PBS
Jha et al. (43)	Range 6–12	38/NA	60 min/d*4 d/wk*6 wk	1,440	observer-blinded	Kinect-based virtual reality gaming	DB	I–III	SL	Balance: PBS
Jung et al. (51)	EG:12.80 ± 1.60 CG:12.00 ± 2.53	10/5	45 min/d*3 d/wk*6 wk	810	single-blind	Kinect Video Game Training	DB	I–II	SL	Balance: PBS
Kachmar et al. (54)	Range 5–18 EG:11.5 ± 3.1 CG:10.8 ± 3.3	25/15	20 min/d*4 d/wk* 2 wk	160	NA	Daily training with personalized balance games	DB	I–III	SL	Balance: TUG/DBT
Lazzari et al. (55)	Mean:7.5 ± 2	20/14	20 min/d*5 d/wk*2 wk	200	double-blind	VR training	DB	I–III	SL	Balance: TUG/PBS
Ledebt et al. (48)	Mean:7.37	10/NA	30 min/d*3 d/wk*6 wk	540	NA	Force plate with real-time feedback with red dot on screen	SB	I	SL	Balance: CoP
Park et al. (60)	Range 6–18 EG:14.3 ± 4.2 CG:14.1 ± 4.3	20/7	40 min/d*2 d/wk*4 wk	320	NA	VR Wii Fit game	DB	III–IV	SL	Balance: MFRT
Pin and Butler (42)	EG:8.92 ± 2.25 CG:9.59 ± 1.87	18/11	20 min/d*4 d/wk*6 wk	480	single-blind	Interactive computer play	DB	III–IV	SL	Balance: PRT
Sahin (9)	Range 7–16 EG:10.5 ± 3.62 CG:10.06 ± 3.24	60/37	45 min/d*2 d/wk*8 wk	720	single-blind	VR intervention	DB	I–III	SL	Balance: BOTMP
Sajan et al. (41)	EG:10.6 ± 3.78 CG:12.4 ± 4.93	20/11	45 min/d*6 d/wk*3 wk	810	single-blind	Wii Games	SB/DB	I–IV	SL	Balance: CoP/PBS
Tarakci et al. (56)	EG:10.46 ± 2.69 CG:10.53 ± 2.79	30/19	20 min/d*2 d/wk*12 wk	480	single-blind	Nintendo Wii-Fit(R) video games	DB	I–III	SL	Balance: FRT/TUG
Uysal and Baltaci (57)	Range 6–14	24/10	30 min/d*2 d/wk*12 wk	720	single-blind	Nintendo Wii (TM) Training	DB	I–III	SL	Balance: PBS

*PBS, Pediatric Balance Scale; FRT, Functional Reach Test; MFRT, Modified Functional Reach Test; TUG, Timed Up and Go Test; BOTMP, Bruininks-Oseretsky Test of Motor Proficiency; CoP, CoP Kinematics; MABC-2, Movement Assessment Battery for Children-2; DBT, Dynamic Balance Test; PRT, Pediatric Reach Test; SL, supervised location*.

*SB, static balance; DB, dynamic balance*.

### PEDro Scale Outcomes

Sixteen studies included in the meta-analysis identified PEDro scores to assess methodological quality. Of these studies, fourteen scored ≥6 on the PEDro scale, which denotes good quality. The other two studies scored 5 (53) and 3 (58), respectively. The raw scores are shown in Table 2.

**Table 2:** Assessment of quality of study design using PEDro.

**References**	**1**	**2**	**3**	**4**	**5**	**6**	**7**	**8**	**9**	**10**	**11**	**Total**
AlSaif and Alsenany (58)	yes	1	0	1	0	0	0	0	0	0	1	3
Chen et al. (49)	yes	1	0	1	0	0	0	1	1	1	1	6
Cho et al. (52)	yes	1	0	1	0	0	1	1	1	1	1	7
Decavele et al. (59)	yes	1	1	0	0	0	0	1	1	1	1	6
Gatica-Rojas et al. (50)	yes	1	1	1	0	0	0	1	1	1	1	7
Hsieh (53)	yes	1	0	0	0	0	0	1	1	1	1	5
Jha et al. (43)	yes	1	1	1	1	0	0	1	1	1	1	8
Jung et al. (51)	yes	1	1	0	1	1	0	1	1	1	1	8
Kachmar et al. (54)	yes	1	1	1	1	0	0	1	1	1	1	8
Lazzari et al. (55)	yes	1	1	1	1	1	1	1	1	1	1	10
Park et al. (60)	yes	1	0	1	0	0	0	1	1	1	1	6
Pin and Butler (42)	yes	1	1	1	0	0	1	1	1	0	1	7
Sahin (9)	yes	1	1	1	1	1	0	1	1	1	1	9
Sajan et al. (41)	yes	1	1	1	0	0	1	1	1	1	1	8
Tarakci et al. (56)	yes	1	1	1	0	0	0	1	1	1	1	7
Uysal and Baltaci (57)	yes	1	1	1	1	0	0	1	1	1	1	8

### Virtual Reality Intervention Setup

**Figure 3:**
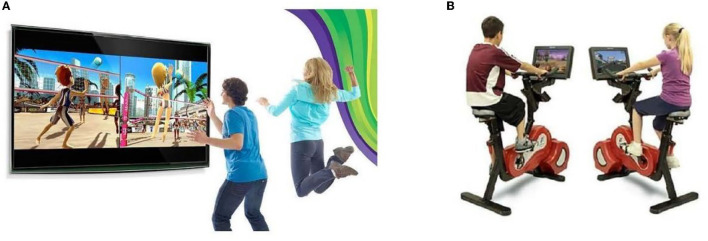
Virtual scenario settings [**(A)** Game training; **(B)** Interactive exercise].

The applied VR interventions could be categorized into two groups (Figure 3). One group of 11 studies used the intervention with a game component (19, 41, 42, 50, 52, 54, 56–60), and the other seven used real-time feedback or interactive exercise in the virtual world (9, 43, 48, 49, 51, 53, 55). The interventions in all these studies were as follows: the total amount of treatment (160–1,680 min), duration of treatment (20–60 min), and intervention period (2–12 weeks). Furthermore, the treatment location varied across studies: for example, some used VR sessions supervised by researchers or therapists, while in other studies, participants practiced at home, and no studies combined both for home exercise.

### Outcome Measure of Balance

Of the 18 studies that used balance as an outcome measure, five reported static balance (Table 1). Ledebt et al. (48), Arnoni et al. (19), Gatica-Rojas et al. (50), and Hsieh et al. (53) used static balance on a force platform; After using VR, four studies found a significant improvement between the intervention and control groups, whereas Sajan et al. did not find a significant improvement between groups (41).

For dynamic balance, there were various tests used in 15 articles. Cho et al. (52), Decavele et al. (59), Jung et al. (51), Hsieh et al. (53), and Uysal et al. (57) used PBS in RCTs and found significant improvement in balance compared to the control group in the intervention group, while Jha et al. (43), Sajn et al. (41) found no significant improvement. Tarakci et al. used two performance scales (FRT & TUG) to test dynamic balance and found a significant difference in improvement between the control and VR intervention groups (56). Park et al. further confirmed the effect of the intervention using the MFRT (60). Hsieh et al. (53), Kachmar et al. (54), and Lazzari et al. (55) also used TUG to confirm further significant improvement. AlSaif et al. also confirmed a significant effect of the Nintendo Wii intervention using the MABC-2 test (58). However, Pin et al. did not find a significant difference in improvement between the control and intervention groups using PRT (42). Chen et al. did not find a significant difference in improvement between the control and intervention groups using BOTMP (49), but Sahin et al. experimentally indicated that the Kinect- based VR intervention was significant (9). Of the included 18 studies, fourteen showed significant effectiveness of applied VR interventions. Moreover, five articles used both methods for outcome measurement (9, 53–56). Of these, two measured dynamic and static balance outcomes (41, 53), and three were focused on dynamic balance (54–56). Sajan et al.'s study showed no significant difference between the experimental and control groups for either dynamic balance or static balance (41).

### Meta-Analysis

**Figure 4:**
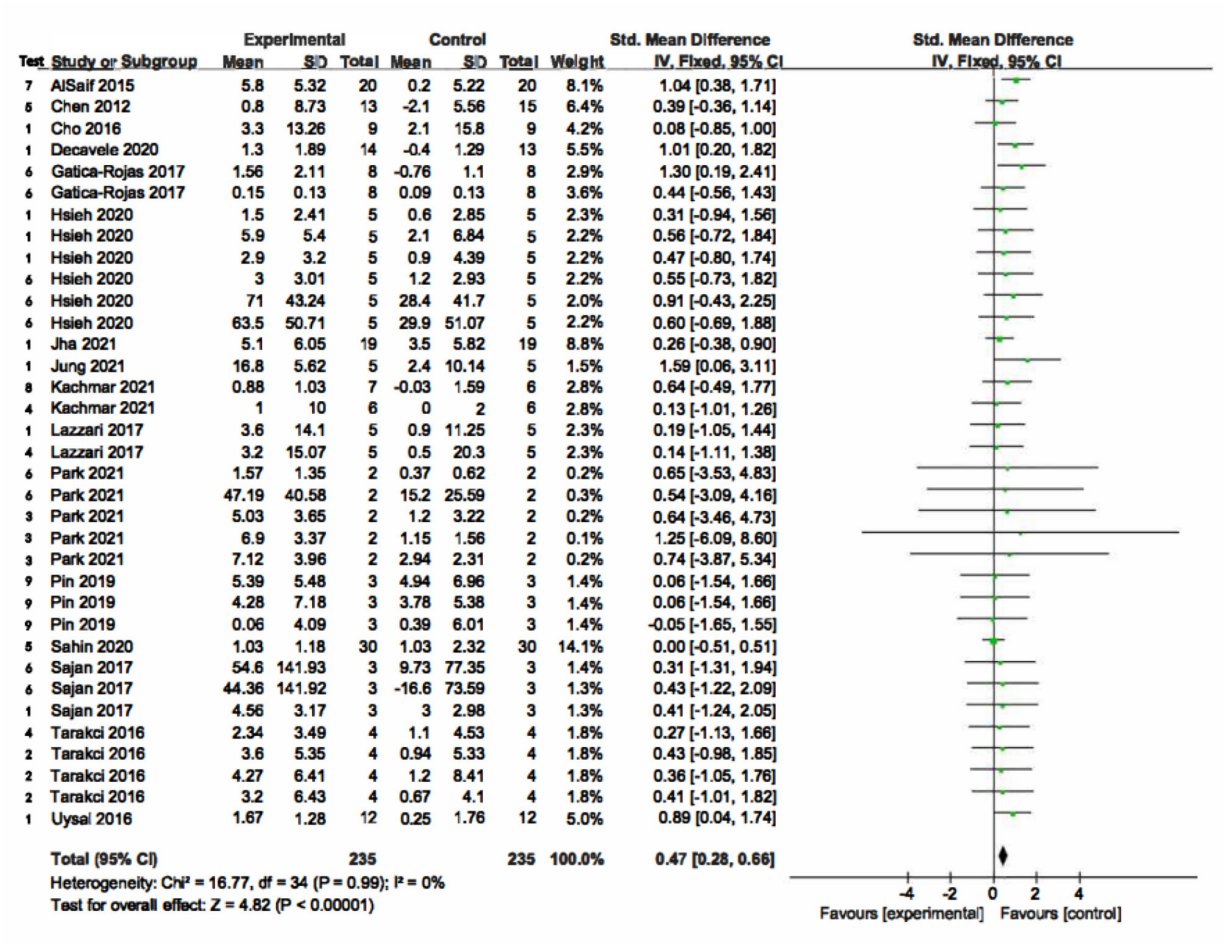
The results of Meta-analysis balance. In the “Test” column, 1 = PBS (Pediatric Balance Scale); 2 = FRT (Functional Reach Test); 3 = MFRT (Modified Functional Reach Test); 4 = TUG (Timed Up and Go Test); 5 = BOTMP (Bruininks-Oseretsky Test of Motor Proficiency); 6 = CoP Kinematics; 7 = MABC-2 (Movement Assessment Battery for Children-2); 8 = DBT (Dynamic Balance Test); 9 = PRT (Pediatric Reach Test).

The meta-analysis of balance included 16 of 18 RCTs studies, and non-inclusion of two studies was due to unavailability of data. The outcome measures were BOTMP, MABC-2, PBS, TUG, FRT (MFRT), PRT, DBT, and CoP. There were 235 participants in the VR group and 235 participants in the control group. The combined statistics was not heterogeneous (*x*^2^ = 16.77, *df* = 34, *p* = 0.99, I^2^ = 0%). The SMD value was 0.47 [95% CI, SD 0.28, 0.66] supporting the VR intervention (Figure 4). The effect size of meta-analysis was moderate using Cohen's 1988 Cochrane Handbook rules (47).

## Discussion

This systematic review and meta-analysis showed a significant improvement in balance for children with CP after VR therapy. As a result of this intervention effect, we believe the VR therapy is a well-designed intervention that can be used as a complementary therapy during the rehabilitation of children with CP. The findings of this RCT review study are consistent with previous studies on VR therapy for children with CP (38, 46, 61).

The study included high-quality literature, and there was no heterogeneity in the results measuring balance ability. These findings were not consistent with the high heterogeneity in balance ability in the previous study (38). Warnier et al. concluded that test diversity contributed to the high heterogeneity in the combined effect (38). In contrast, the present study included literature that used more measurement instruments. It may include the nine most recent publications from 2019-2021 in this study (9, 19, 42, 43, 51, 53, 54, 59, 60). VR treatment studies have become increasingly popular in recent years, with many recent publications (62–65). However, the quality of included RCT studies is generally high. It is supported by PEDro methodological quality assessment. This study included studies with a score of 6 and above in 87.5%, compared to only 57.1% in the Warnier et al.'s studies (38).

Nevertheless, there are many methodological limitations to the study in this article, and the results should be interpreted with caution in practical applications. For example, there are many doubts regarding exercise duration and the exercise effect (66). The total time of exercise is theoretically proportional to the exercise effect, and prolonged exercise is expected to yield good exercise benefits (67). However, in the studies by Chen et al. (49) and Jha et al. (43), the total exercise duration of VR amounted to 1,440 min, but it did not produce the expected intervention effect. In contrast, the studies of Kachmar et al. (54), Lazzari et al. (55), and Park et al. (60) showed positive effects within the exercise duration of only 160–320 min. Therefore, the relationship between VR exercise duration and exercise benefits needs to be further explored.

In addition, the principles regarding the use of dynamic and static balance in the balance test are opposite. In this study, the difference data from the static balance test results are reversed and used in the meta-analysis. Also, the absence of differential data from the original study text included in the study and the differential data obtained from indirect calculations may lead to bias in the results (68). The unavailability of data from two studies that were not included in the study may also affect the meta-analysis results (19, 48). Nevertheless, four of the included studies had findings that did not support the view that VR treatment was more effective than the other traditional treatments (41–43, 49). Despite the popularity of VR therapy as a newly popular adjunctive technology, we should use it with caution in clinical practice in the absence of a clearly defined consensus (69).

Based on the above limitations, future studies should focus on experimental design, increasing the number of participants, standardizing measurement criteria, clarifying interventions for VR, and strict randomized controlled trial processes to provide more valid evidence for VR treatment. Furthermore, several studies on the role of VR in the management of children with CP (70), the use of robotics in the neuromotor rehabilitation of children with CP (71), and the use of hybrid assisted limb (HAL) for robot-assisted gait training of cerebral palsy patients (72) all contributed to the future direction of VR motor rehabilitation for children with CP.

## Conclusion

Preliminary evidence indicates that VR therapy has a positive effect on improving balance function in children with CP. At least 20 min per session, twice a week for six weeks or more of regular VR therapy is more effective for improving balance function in children with CP. The application of robotics in motor function will be the new direction of VR therapy for children with CP in the future (73).

## Data Availability Statement

The original contributions presented in the study are included in the article/supplementary material, further inquiries can be directed to the corresponding authors.

## Author Contributions

WL, YH, and JL: data collection. WL and YH: data analysis, conception, and design. WL, YH, JL, and JC: research design, writing the manuscript and revision. All authors contributed to the article and approved the submitted version.

## Funding

This study was funded by the Humanities and Social Sciences Project of the Ministry of Education of China (No. 17YJC890020).

## Conflict of Interest

The authors declare that the research was conducted in the absence of any commercial or financial relationships that could be construed as a potential conflict of interest.

## Publisher's Note

All claims expressed in this article are solely those of the authors and do not necessarily represent those of their affiliated organizations, or those of the publisher, the editors and the reviewers. Any product that may be evaluated in this article, or claim that may be made by its manufacturer, is not guaranteed or endorsed by the publisher.

## References

[1] Yu L Yan L Chen M Dong L. Cyclostationary modeling of surface electromyography signal during gait cycles and its application for cerebral palsy diagnosis. J Shanghai Jiaotong Univ. (2018) 1:56–61. 10.1007/s12204-018-2023-9

[2] Ren Z Wu J. The Effect of Virtual Reality Games on the Gross Motor Skills of Children with Cerebral Palsy: a Meta-Analysis of Randomized Controlled Trials. Int J Environ Res Public Health. (2019) 20:3885. 10.3390/ijerph16203885 31614990 PMC6843701

[3] Amankwah N Oskoui M Garner R Bancej C Manuel DG Wall R . Cerebral palsy in Canada, 2011-2031: results of a microsimulation modelling study of epidemiological and cost impacts. Health Promot Chronic Dis Prev Can. (2020) 2:25–37. 10.24095/hpcdp.40.2.01 32049464 PMC7053851

[4] LI X Qiu H Jiang Z Pang W Guo J Zhu L . Epidemiological characteristics of cerebral palsy in twelve province in China. Chin J Appl Clin Pediatr. (2018) 5:378–83.

[5] Yang S Xia J Gao J Wang L. Increasing prevalence of cerebral palsy among children and adolescents in China 1988-2020: a systematic review and meta-analysis. J Rehabil Med. (2021) 5:jrm00195. 10.2340/16501977-2841 33961057 PMC8814846

[6] Bilde PE Kliim-Due M Rasmussen B Petersen LZ Petersen TH Nielsen JB. Individualized, home-based interactive training of cerebral palsy children delivered through the Internet. BMC Neurol. (2011) 32. 10.1186/1471-2377-11-32 21392370 PMC3061895

[7] Gormley ME Jr. Treatment of neuromuscular and musculoskeletal problems in cerebral palsy. Pediatr Rehabil. (2001) 1:5–16. 10.1080/13638490151068393 11330850

[8] Chang J Li Y Song H Yong L Luo L Zhang Z . Assessment of Validity of Children's Movement Skill Quotient (CMSQ) Based on the Physical Education Classroom Environment. Biomed Res Int. (2020) 8938763. 10.1155/2020/8938763 33123588 PMC7586154

[9] Sahin S Köse B Aran OT Bahadir Agce Z Kayihan H. The Effects of Virtual Reality on Motor Functions and Daily Life Activities in Unilateral Spastic Cerebral Palsy: a Single-Blind Randomized Controlled Trial. Games Health J. (2020) 1:45–52. 10.1089/g4h.2019.0020 31335174

[10] Farhat F Hsairi I Baati H Smits-Engelsman BC Masmoudi K Mchirgui R . The effect of a motor skills training program in the improvement of practiced and non-practiced tasks performance in children with developmental coordination disorder (DCD). Hum Mov Sci. (2016) 46:10–22. 10.1016/j.humov.2015.12.001 26703915

[11] Coq JO Kochmann M Lacerda DC Khalki H Delcour M Toscano AE . From cerebral palsy to developmental coordination disorder: Development of preclinical rat models corresponding to recent epidemiological changes. Ann Phys Rehabil Med. (2020) 5:422–30. 10.1016/j.rehab.2019.10.002 31756523

[12] Tinderholt Myrhaug H Østensjø S Larun L Odgaard-Jensen J Jahnsen R. Intensive training of motor function and functional skills among young children with cerebral palsy: a systematic review and meta-analysis. BMC Pediatr. (2014) 14:292. 10.1186/s12887-014-0292-5 25475608 PMC4265534

[13] Brandão MB Ferre C Kuo HC Rameckers EA Bleyenheuft Y Hung YC . Comparison of Structured Skill and Unstructured Practice During Intensive Bimanual Training in Children With Unilateral Spastic Cerebral Palsy. Neurorehabil Neural Repair. (2014) 5:452–61. 10.1177/1545968313516871 24376067

[14] Nielsen JB Cohen LG. Does corticospinal plasticity play a role in acquisition of skills required for high-performance sports? J Physiol. (2008) 1:65–70. 10.1113/jphysiol.2007.142661 17717010 PMC2375560

[15] Ryan JM Cassidy EE Noorduyn SG O'Connell NE. Exercise interventions for cerebral palsy. Cochrane Database Syst Rev. (2017) 6:CD011660. 10.1002/14651858.CD011660.pub2 28602046 PMC6481791

[16] Jensen JL Marstrand PC Nielsen JB. Motor skill training and strength training are associated with different plastic changes in the central nervous system. J Appl Physiol (1985). (2005) 99:1558–68. 10.1152/japplphysiol.01408.2004 15890749

[17] Dodd KJ Taylor NF Graham HK. A randomized clinical trial of strength training in young people with cerebral palsy. Dev Med Child Neurol. (2003) 10:652–7. 10.1017/S0012162203001221 14515935

[18] Douglas MP Ladabaum U Pletcher MJ Marshall DA Phillips KA. Economic evidence on identifying clinically actionable findings with whole-genome sequencing: a scoping review. Genet Med. (2016) 2:111–6. 10.1038/gim.2015.69 25996638 PMC4654986

[19] Arnoni JLB Pavão SL Dos Santos Silva FP Rocha NACF. Effects of virtual reality in body oscillation and motor performance of children with cerebral palsy: A preliminary randomized controlled clinical trial. Complement Ther Clin Pract. (2019) 35:189–94. 10.1016/j.ctcp.2019.02.014 31003657

[20] Juneja M Jain R Gautam A Khanna R Narang K. Effect of multilevel lower-limb botulinum injections & intensive physical therapy on children with cerebral palsy. Indian J Med Res. (2017) 146:S8–S14. 10.4103/ijmr.IJMR_1223_15 29578189 PMC5890601

[21] Katz-Leurer M Rotem H Keren O Meyer S. The effects of a 'home-based' task-oriented exercise programme on motor and balance performance in children with spastic cerebral palsy and severe traumatic brain injury. Clin Rehabil. (2009) 8:714–24. 10.1177/0269215509335293 19506005

[22] Türker D Korkem D Özal C Günel MK Karahan S. The effects of neurodevelopmental (Bobath) therapy based goal directed therapy on gross motor function and functional status of children with cerebral palsy. Int J Therap Rehabil Res. (2015) 4:9–20. 10.5455/ijtrr.00000060

[23] Yazmir B Reiner M. Monitoring brain potentials to guide neurorehabilitation of tracking impairments. IEEE Int Conf Rehabil Robot. (2017) 2017:983–8. 10.1109/ICORR.2017.8009377 28813949

[24] Ashkenazi T Laufer Y Orian D Weiss P L. Effect of training children with Developmental Coordination Disorders in a virtual environment compared with a conventional environment. In: 2013 International Conference on Virtual Rehabilitation (ICVR). Philadelphia, PA: IEEE. (2013). pp. 46–50.

[25] Snider L Majnemer A Darsaklis V. Virtual reality as a therapeutic modality for children with cerebral palsy. Dev Neurorehabil. (2010) 2:120–8. 10.3109/17518420903357753 20222773

[26] Tatla SK Sauve K Virji-Babul N Holsti L Butler C Van Der Loos HF. Evidence for outcomes of motivational rehabilitation interventions for children and adolescents with cerebral palsy: an American Academy for Cerebral Palsy and Developmental Medicine systematic review. Dev Med Child Neurol. (2013) 7:593–601. 10.1111/dmcn.12147 23550896

[27] Gordon C Roopchand-Martin S Gregg A. Potential of the Nintendo Wii™ as a rehabilitation tool for children with cerebral palsy in a developing country: a pilot study. Physiotherapy. (2012) 3:238–42. 10.1016/j.physio.2012.05.011 22898581

[28] Luna-Oliva L Ortiz-Gutiérrez RM Cano-de la Cuerda R Piédrola RM Alguacil-Diego IM Sánchez-Camarero C . Kinect Xbox 360 as a therapeutic modality for children with cerebral palsy in a school environment: a preliminary study. NeuroRehabilitation. (2013) 4:513–21. 10.3233/NRE-131001 24018364

[29] Ustinova KI Perkins J Szostakowski L Tamkei LS Leonard WA. Effect of viewing angle on arm reaching while standing in a virtual environment: potential for virtual rehabilitation. Acta Psychol (Amst). (2010) 2:180–90. 10.1016/j.actpsy.2009.11.006 20021998

[30] Deutsch JE Mirelman A. Virtual reality-based approaches to enable walking for people poststroke. Top Stroke Rehabil. (2007) 6:45–53. 10.1310/tsr1406-45 18174115

[31] Shin JW Song GB Hwangbo G. Effects of conventional neurological treatment and a virtual reality training program on eye-hand coordination in children with cerebral palsy. J Phys Ther Sci. (2015) 7:2151–4. 10.1589/jpts.27.2151 26311943 PMC4540838

[32] Brien M Sveistrup H. An intensive virtual reality program improves functional balance and mobility of adolescents with cerebral palsy. Pediatr Phys Ther. (2011) 3:258–66. 10.1097/PEP.0b013e318227ca0f 21829120

[33] Harris K Reid D. The influence of virtual reality play on children's motivation. Can J Occup Ther. (2005) 1:21–9. 10.1177/000841740507200107 15727045

[34] Chen YP Lee SY Howard AM. Effect of virtual reality on upper extremity function in children with cerebral palsy: a meta-analysis. Pediatr Phys Ther. (2014) 3:289–300. 10.1097/PEP.0000000000000046 24819682

[35] Chen Y Garcia-Vergara S Howard AM. Effect of a Home-Based Virtual Reality Intervention for Children with Cerebral Palsy Using Super Pop VR Evaluation Metrics: a Feasibility Study. Rehabil Res Pract. (2015) 2015:812348. 10.1155/2015/812348 26457202 PMC4589626

[36] Pavão SL Arnoni JLB Rocha NACF. Effects of Visual Manipulation in Sit-to-Stand Movement in Children With Cerebral Palsy. J Mot Behav. (2018) 5:486–91. 10.1080/00222895.2017.1367641 28976286

[37] Fehlings D Switzer L Findlay B Knights S. Interactive computer play as “motor therapy” for individuals with cerebral palsy. Semin Pediatr Neurol. (2013) 2:127–38. 10.1016/j.spen.2013.06.003 23948687

[38] Warnier N Lambregts S. Port IV Effect of Virtual Reality Therapy on Balance and Walking in Children with Cerebral Palsy: a systematic review. Dev Neurorehabil. (2020) 8:502–18. 10.1080/17518423.2019.1683907 31674852

[39] Ravi DK Kumar N Singhi P. Effectiveness of virtual reality rehabilitation for children and adolescents with cerebral palsy: an updated evidence-based systematic review. Physiotherapy. (2017) 3:245–58. 10.1016/j.physio.2016.08.004 28109566

[40] Kilgour G Adair B Stott NS Steele M Hogan A Imms C. Do physical activity interventions influence subsequent attendance and involvement in physical activities for children with cerebral palsy: a systematic review. Disabil Rehabil. (2021) 7:1–17. 10.1080/09638288.2021.1909151 34097836

[41] Sajan JE John JA Grace P Sabu SS Tharion G. Wii-based interactive video games as a supplement to conventional therapy for rehabilitation of children with cerebral palsy: a pilot, randomized controlled trial. Dev Neurorehabil. (2017) 6:361–7. 10.1080/17518423.2016.1252970 27846366

[42] Pin TW Butler PB. The effect of interactive computer play on balance and functional abilities in children with moderate cerebral palsy: a pilot randomized study. Clin Rehabil. (2019) 4:704–10. 10.1177/0269215518821714 30599772

[43] Jha KK Karunanithi GB Sahana A Karthikbabu S. Randomised trial of virtual reality gaming and physiotherapy on balance, gross motor performance and daily functions among children with bilateral spastic cerebral palsy. Somatosens Mot Res. (2021) 2:117–26. 10.1080/08990220.2021.1876016 33655813

[44] Liberati A Altman DG Tetzlaff J Mulrow C Gøtzsche PC Ioannidis JP . The PRISMA statement for reporting systematic reviews and meta-analyses of studies that evaluate health care interventions: explanation and elaboration. Ann Intern Med. (2009) 4:W65–94. 10.7326/0003-4819-151-4-200908180-00136 19622512

[45] Moseley AM Herbert RD Sherrington C Maher CG. Evidence for physiotherapy practice: a survey of the Physiotherapy Evidence Database (PEDro). Aust J Physiother. (2002) 1:43–9. 10.1016/S0004-9514(14)60281-6 11869164

[46] Wu J Loprinzi PD Ren Z. The Rehabilitative Effects of Virtual Reality Games on Balance Performance among Children with Cerebral Palsy: A Meta-Analysis of Randomized Controlled Trials. Int J Environ Res Public Health. (2019) 21:41–61. 10.3390/ijerph16214161 31661938 PMC6861947

[47] Cohen J. Statistical Power Analysis for the Behavioral Sciences. Cambridge, MA: Academic press. (2013).

[48] Ledebt A Becher J Kapper J Rozendaalr RM Bakker R Leenders IC . Balance training with visual feedback in children with hemiplegic cerebral palsy: effect on stance and gait. Motor Control. (2005) 4:459–68. 10.1123/mcj.9.4.459 16333148

[49] Chen CL Hong WH Cheng HY Liaw MY Chung CY Chen CY. Muscle strength enhancement following home-based virtual cycling training in ambulatory children with cerebral palsy. Res Dev Disabil. (2012) 4:1087–94. 10.1016/j.ridd.2012.01.017 22502833

[50] Gatica-Rojas V Méndez-Rebolledo G Guzman-Muñoz E Soto-Poblete A Cartes-Velásquez R Elgueta-Cancino E . Does Nintendo Wii Balance Board improve standing balance? A randomized controlled trial in children with cerebral palsy. Eur J Phys Rehabil Med. (2017) 4:535–44. 10.23736/S1973-9087.16.04447-6 27882910

[51] Jung S Song S Lee D Lee K Lee G. Effects of Kinect Video Game Training on Lower Extremity Motor Function, Balance, and Gait in Adolescents with Spastic Diplegia Cerebral Palsy: a pilot randomized controlled trial. Dev Neurorehabil. (2021) 3:159–65. 10.1080/17518423.2020.1819458 32981401

[52] Cho C Hwang W Hwang S Chung Y. Treadmill Training with Virtual Reality Improves Gait, Balance, and Muscle Strength in Children with Cerebral Palsy. Tohoku J Exp Med. (2016) 3:213–8. 10.1620/tjem.238.213 26947315

[53] Hsieh HC. Preliminary Study of the Effect of Training With a Gaming Balance Board on Balance Control in Children With Cerebral Palsy: A Randomized Controlled Trial. Am J Phys Med Rehabil. (2020) 2:142–8. 10.1097/PHM.0000000000001300 31464757

[54] Kachmar O Kushnir A Fedchyshyn B Cristiano J O'Flaherty J Helland K . Personalized balance games for children with cerebral palsy: A pilot study. J Pediatr Rehabil Med. (2021) 14:237–45. 10.3233/PRM-190666 33720857

[55] Lazzari RD Politti F Belina SF Collange Grecco LA Santos CA Dumont AJL . Effect of Transcranial Direct Current Stimulation Combined With Virtual Reality Training on Balance in Children With Cerebral Palsy: a Randomized, Controlled, Double-Blind, Clinical Trial. J Mot Behav. (2017) 3:329–36. 10.1080/00222895.2016.1204266 27644454

[56] Tarakci D Ersoz Huseyinsinoglu B Tarakci E Razak Ozdincler A. Effects of Nintendo Wii-Fit® video games on balance in children with mild cerebral palsy. Pediatr Int. (2016) 10:1042–50. 10.1111/ped.12942 26858013

[57] Atasavun Uysal S Baltaci G. Effects of Nintendo Wii™ Training on Occupational Performance, Balance, and Daily Living Activities in Children with Spastic Hemiplegic Cerebral Palsy: a Single-Blind and Randomized Trial. Games Health J. (2016) 5:311–7. 10.1089/g4h.2015.0102 27705006

[58] AlSaif AA Alsenany S. Effects of interactive games on motor performance in children with spastic cerebral palsy. J Phys Ther Sci. (2015) 6:2001–3. 10.1589/jpts.27.2001 26180367 PMC4500030

[59] Decavele S Ortibus E Van Campenhout A Molenaers G Jansen B Omelina L . The Effect of a Rehabilitation Specific Gaming Software Platform to Achieve Individual Physiotherapy Goals in Children with Severe Spastic Cerebral Palsy: a Randomized Crossover Trial. Games Health J. (2020) 9:376–85. 10.1089/g4h.2019.0097 32614723

[60] Park SH Son SM Choi JY. Effect of posture control training using virtual reality program on sitting balance and trunk stability in children with cerebral palsy. NeuroRehabilitation. (2021) 3:247–54. 10.3233/NRE-201642 33843705

[61] Cassani R Novak GS Falk TH Oliveira AA. Virtual reality and non-invasive brain stimulation for rehabilitation applications: a systematic review. J Neuroeng Rehabil. (2020) 1:147. 10.1186/s12984-020-00780-5 33129331 PMC7603766

[62] Arnoni JLB Kleiner AFR Lima CRG de Campos AC Rocha NACF. Nonimmersive Virtual Reality as Complementary Rehabilitation on Functional Mobility and Gait in Cerebral Palsy: A Randomized Controlled Clinical Trial. Games Health J. (2021) 4:254–63. 34370612 10.1089/g4h.2021.0009

[63] Cheng M Anderson M Levac DE. Performance Variability During Motor Learning of a New Balance Task in a Non-immersive Virtual Environment in Children With Hemiplegic Cerebral Palsy and Typically Developing Peers. Front Neurol. (2021) 12:623200. 10.3389/fneur.2021.623200 33790848 PMC8005528

[64] Beani E Filogna S Martini G Barzacchi V Ferrari A Guidi E . Application of Virtual Reality Rehabilitation System for the assessment of postural control while standing in typical children and peers with neurodevelopmental disorders. Gait Posture. (2021) 92:364–70. 10.1016/j.gaitpost.2021.12.008 34923256

[65] Amirthalingam J Paidi G Alshowaikh K Iroshani Jayarathna A Salibindla DBAMR Karpinska-Leydier K . Virtual Reality Intervention to Help Improve Motor Function in Patients Undergoing Rehabilitation for Cerebral Palsy, Parkinson's Disease, or Stroke: a Systematic Review of Randomized Controlled Trials. Cureus. (2021) 7:e16763. 10.7759/cureus.16763 34367835 PMC8343554

[66] Bonnechère B Omelina L Jansen B Van Sint Jan S. Balance improvement after physical therapy training using specially developed serious games for cerebral palsy children: preliminary results. Disabil Rehabil. (2017) 4:403–6. 10.3109/09638288.2015.1073373 28033958

[67] Zakharova AN Kironenko TA Milovanova KG Orlova AA Dyakova EY Kalinnikova Yu G . Treadmill Training Effect on the Myokines Content in Skeletal Muscles of Mice With a Metabolic Disorder Model. Front Physiol. (2021) 12:709039. 10.3389/fphys.2021.709039 34858197 PMC8631430

[68] Chang J Yong L Yan H Wang J Song N. Measurement Properties of Canadian Agility and Movement Skill Assessment for Children Aged 9–12 Years Using Rasch Analysis. Front Public Health. (2021) 9:745449. 10.3389/fpubh.2021.745449 34938702 PMC8685226

[69] Chaparro-Rico BD Cafolla D. Test-retest, inter-rater and intra-rater reliability for spatiotemporal gait parameters using SANE (an eaSy gAit aNalysis systEm) as measuring instrument. Appl Sci. (2020) 17:5781. 10.3390/app10175781

[70] Weiss PL Tirosh E Fehlings D. Role of virtual reality for cerebral palsy management. J Child Neurol. (2014) 8:1119–24. 10.1177/0883073814533007 24799367

[71] Krebs HI Volpe BT Aisen ML Hening W Adamovich S Poizner H . Robotic applications in neuromotor rehabilitation. Robotica. (2003) 21:3–11. 10.1017/S0263574702004587

[72] Matsuda M Mataki Y Mutsuzaki H Yoshikawa K Takahashi K Enomoto K . Immediate effects of a single session of robot-assisted gait training using Hybrid Assistive Limb (HAL) for cerebral palsy. J Phys Ther Sci. (2018) 2:207–12. 10.1589/jpts.30.207 29545679 PMC5851348

[73] Fridin M Belokopytov M. Robotics Agent Coacher for CP motor Function (RAC CP Fun). Robotica. (2014) 8:1265–79. 10.1017/S026357471400174X

